# Long range Trp-Trp interaction initiates the folding pathway of a pro-angiogenic β-hairpin peptide

**DOI:** 10.1038/srep16651

**Published:** 2015-11-25

**Authors:** Donatella Diana, Lucia De Rosa, Maddalena Palmieri, Anna Russomanno, Luigi Russo, Carmelo La Rosa, Danilo Milardi, Giorgio Colombo, Luca D. D’Andrea, Roberto Fattorusso

**Affiliations:** 1 Istituto di Biostrutture e Bioimmagini, C.N.R., via Mezzocannone 16, 80134, Napoli (Italy); 2 Dipartimento di Scienze e Tecnologie Ambientali, Biologiche e Farmaceutiche, Seconda Università degli Studi Napoli, via Vivaldi 43, 81100, Caserta (Italy); 3 Dipartimento di Scienze Chimiche, Università degli Studi di Catania, viale A. Doria 6, 95125, Catania (Italy); 4 Istituto di Biostrutture e Bioimmagini, C.N.R., Via Gaifami 18, 95126, Catania (Italy); 5 Istituto di Chimica del Riconoscimento Molecolare, C.N.R., via Bianco 9, 20131, Milano (Italy)

## Abstract

HPLW, a designed VEGF (Vascular Endothelium Growth Factor) receptor-binding peptide, assumes a well folded β-hairpin conformation in water and is able to induce angiogenesis *in vivo*. In this study, we investigated at atomic resolution the thermal folding/unfolding pathway of HPLW by means of an original multi-technique approach combining DSC, NMR, MD and mutagenesis analyses. In particular, careful NMR investigation of the single proton melting temperatures together with DSC analysis accurately delineate the peptide folding mechanism, which is corroborated by computational folding/unfolding simulations. The HPLW folding process consists of two main events, which are successive but do not superimpose. The first folding step initiates at 320 K upon the hydrophobic collapse of the Trp5 and Trp13 side-chains which stabilizes the concurrent β-turn formation, whose COi-HNi + 3 hydrogen bond (Asp10 → Arg7) appears particularly stable. At 316 K, once the β-turn is completely formed, the two β-strands pair, very likely starting by Trp5 and Trp13, which thus play a key role also in the final step of the β-hairpin folding. Overall, here we describe a multi-state hierarchical folding pathway of a highly structured β-hairpin, which can be classified as a broken-zipper mechanism.

Protein folding represents one of the most intensively studied phenomena of recent times in biology. Yet, the molecular mechanisms by which a peptide chain reaches its native structure have not been yet fully understood^1^,^2^. Importantly, understanding protein folding pathways plays an essential role in the comprehension of many diseases known to be caused by protein misfolding processes, also considering that every functional protein is permanently in equilibrium with its unfolded state^3^,^4^. As a matter of fact, evolutionary pressure favored protein structures characterized by folding pathways preventing the formation of uncontrolled misfolded states, which in some peculiar conditions, either pathological or physiological, may anyhow occur. A fundamental process in protein folding appears to be the formation of secondary structural elements that make up the native structure. In this context, peptide model systems have been designed and structurally characterized, providing key insights into understanding the relationship between sequence, folded structure and stability^5^ . In model helix peptides structure stabilization is largely a consequence of local interactions. In contrast, β-sheet are propagated by residues in quite distant regions of the peptide sequence. A β-hairpin represents a significant context-free model for examining the nature of β-sheet stabilizing interactions relevant to protein stability and initiation events during folding^6^,^7^,^8^,^9^,^10^,^11^,^12^,^13^,^14^,^15^. Particularly, the frequent observation for β-sheet proteins that one β-hairpin is formed before or at the folding transition state proves the involvement of the hairpin in nucleation-condensation pathway^16^,^17^,^18^,^19^. Nonetheless, while many short peptides have been widely and successfully adopted in probing the stability of α-helical and β-turn structures^20^,^21^,^22^,^23^,^24^,^25^, only a reduced number of sequences have been identified that fold autonomously in water to form monomeric β-hairpins^26^,^27^,^28^,^29^,^30^,^31^,^32^,^33^.

In the last decade, we have designed several α-helix and β-hairpin peptides, able to act as VEGF modulators *in vitro* and *in vivo* essentially thanks to their structural pre-organization^34^,^35^,^36^,^37^,^38^,^39^,^40^. Among them, we reported the structure-based design of a VEGF receptor binding peptide, named HPLW (NH2-Lys-Gln-Leu-Leu-Trp-Ile-Arg-Ser-Gly-Asp-Arg-Pro-Trp-Tyr-Tyr-Thr-Ser-OH), mimicking the PlGF (Placenta Growth Factor) amino acids sequence 87–100^37^. In aqueous solution HPLW assumes a well folded monomeric β-hairpin conformation with high structural similarity with the natural sequence. Particularly, HPLW folds to a significant degree in water without the need of non-natural amino acids or disulphide bonds incorporation. Furthermore, the molecular determinants of HPLW ability to bind the VEGF receptors have been determined both *in vitro* and in a cellular environment^37^,^41^.

In this study, we investigated at atomic resolution the thermal folding/unfolding pathway of HPLW peptide by means of DSC (Differential Scanning Calorimetry), NMR (Nuclear Magnetic Resonance) and MD (Molecular Dynamics) methodologies. A hierarchical sequence of folding events has been outlined and further confirmed by the high-resolution determination of HPLW structure at 318 K, just in between the two main folding transitions. Finally, we have analyzed the structural features of HPLW designed mutants to better clarify the relationship between HPLW sequence and β-hairpin folding pathway and stability.

## Results

### DSC analysis of HPLW thermal unfolding

**Figure 1:**
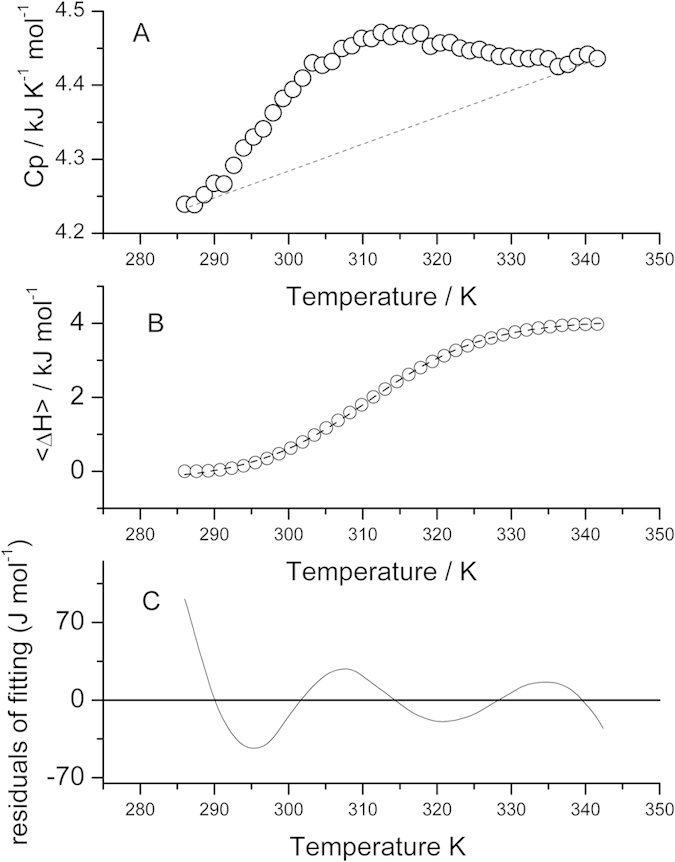
Panel **(A)** thermal unfolding profile of HPLW monitored by DSC (open circles). The dotted line represents a linear interpolation of the onset and offset points. The temperature of maximum Cp value (Tm) is 316.4 K. Panel (**B**) excess enthalpy obtained by integrating the excess heat capacity curve as described in the text. The dashed line represent the best fitting of the experimental points by the equation: Y = ΔH_cal_(1 − 1/(1 + exp(T − T_1/2_))), where T_1/2_ is the temperature at which the calorimetric enthalpy reaches the 50% of its final value. Panel (**C**) residuals of the curve fitting of the enthalpy vs T curve reported in panel (**B**).

HPLW showed no tendency to aggregate in any of the conditions that we have tested, including peptide concentrations up to the millimolar range. DSC analysis, shown in Fig. 1, revealed that thermally-induced unfolding of this protein is reversible. Indeed, thermograms of a series of up to eight heating-cooling cycles of HPLW indicated a very small decrease in the amplitude throughout multiple scans, as one would expect for accompanying aggregation or any other irreversible phenomena. The van’t Hoff enthalpy ΔH^VH^ was calculated from the calorimetric profiles according to equation (1),




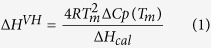




where T_m_, ΔC_p_(Tm) and ΔH_cal_ are the transition temperature, the corresponding excess heat capacity and the calorimetric enthalpy determined from the heat capacity curve by the area of the heat adsorption peak. The ratio of the van’t Hoff to the calorimetric enthalpy (r^VH^ = ΔH^VH^/ΔH_cal_) is known to be a measure of the validity of the assumption that protein unfolding is a two state transition. If these two enthalpies converge to the same value, the implication is that the two-state assumption holds. Indeed, for thermal unfolding of small compact globular proteins this ratio is usually fairly close to 1.0. For HPLW, the calorimetric parameters are: Tm = 316.4 ± 0.1 K, ΔH^VH^ = 127.1 ± 9.3 kJ mol^−1^, ΔH_cal_ = 3.9 ± 0.7 kJ mol^−1^ and the van’t Hoff ratio is r^VH^ = 32, thus suggesting an unfolding mechanism for this peptide rather far from a two-state approximation. The unusually high value of the vant’Hoff ratio found for HPLW could to some extent be due to the uncertainties in baseline subtraction from raw calorimetric peaks (unavoidable in such broad transitions), which decrease the value of calorimetric enthalpy thus leading to an increase of the vant’Hoff ratio^42^,^43^,^44^. Nevertheless, the high value of r^VH^ here determined can be reconciled with a non-cooperative peptide thermal unfolding characterized by a weak and very broad calorimetric peak. This behavior is in striking contrast with that observed for most of globular proteins where the large number of intramolecular residue-residue contacts significantly contributes to the cooperativity of the folding process.

### HPLW thermal folding/unfolding analysis and structural characterization at 318 K by NMR spectroscopy

**Figure 2:**
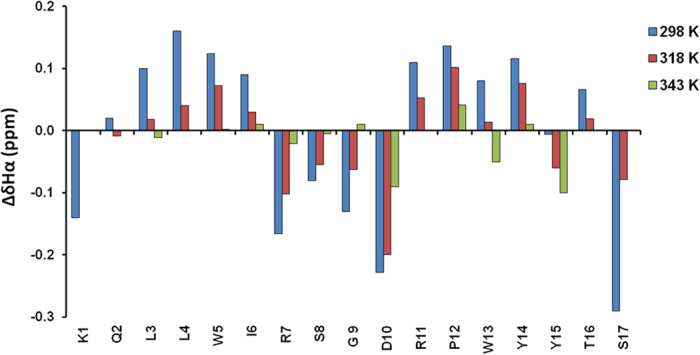
Deviation of Hα chemical shifts of HPLW hairpin from random-coil values (ΔδHα) at 298, 318 and 303 K in water solution at pH 6.8.

**Figure 3:**
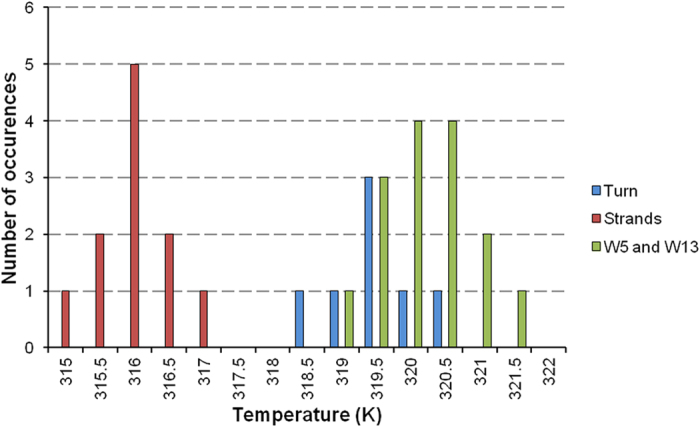
Histogram of T_m_ values for turn protons (blue), strand protons (red) and W5 and W13 protons (green) of HPLW.

To get high resolution structural characterization of HPLW β-hairpin folding pathway, temperature structural changes have been investigated by NMR spectroscopy. In particular, a series of one-dimensional ^1^H and bi-dimensional [^1^H, ^1^H] TOCSY spectra have been acquired from 285 K to 343 K, at regular intervals of 5 K. At 343 K, HPLW Hα chemical shifts are mostly close to their random coil values, indicating a substantial lack of peptide secondary structure (Fig. 2). The analysis of the NMR thermal folding/unfolding curves (Figure S1) revealed that many of the single protons could be fitted using a sigmoidal curve, therefore deriving very punctual atomic thermal transitions^45^. In total, we obtained 30 curves (6 HN, 7 Hα and 5 aliphatic side chain and 12 aromatic side chains protons) with different melting temperatures (T_m_s) distributed in the range between 315.6 and 321.5 K. Interestingly, most of the β-strands residues, *i.e.* Leu3, Leu4, Ile6, Pro12, and Tyr14, contain protons presenting very similar T_m_s included between 315.6 and 317.4 K with a mean value of 316.5 ± 0.5 K; differently, protons of the residues located within the β-turn, Arg7, Ser8, Gly9, Asp10, and of the Trp5 and Trp13 constituting the β-strands, show comparable significantly higher T_m_s, respectively between 318.4 and 320.4 and 319.1 and 321.3 K, with mean values of 319.3 ± 0.3 and 320.1 ± 0.6 K (Fig. 3). When the melting temperatures are grouped in the three clusters above identified, Gaussian-type behaviors of the T_m_s distributions are observed in all the three cases.

**Figure 4:**
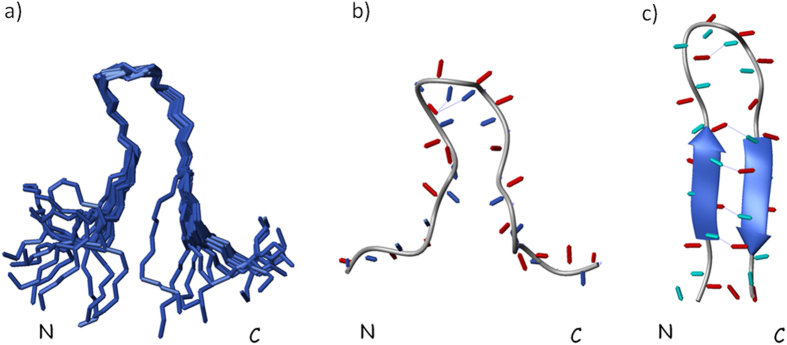
(**a**) Superposed backbone traces for the NMR-derived structural ensemble of HPLW at 318 K. HPLW representative structure (**b**) at 318 K and (**c**) at 298 K. Intra-molecular hydrogen bonds are indicated as blue dotted lines.

To obtain a more resolved structural description of HPLW unfolding process we undertook the peptide structure characterization at 318 K by means of standard NMR^46^. The complete ^1^H assignments (Table S1) at 318 K were obtained after accurate analysis of TOCSY and NOESY spectra. NOESY spectra with different mixing times were acquired to derive NOE linear buildup, which indicated a mixing time of 350 ms as the most appropriate to obtain the distance constraints for structure calculation. At first, the analysis of 2D [^1^H-^1^H]-NOESY provides us with an initial qualitative assessment of the structural changes induced in the peptide by the temperature increase. In contrast to the NOE cross-peaks collected at 298 K, several sequential and long-range NOEs, diagnostic for β-hairpin, disappeared or decreased in intensity at 318 K. In particular, short range NOE connectivities (dαN_(1, 1 + 2)_ NOEs between residues Arg7 and Gly9 and Ser8 and Asp10) were assigned, indicating, accordingly to the ΔδHα analysis, that the HPLW β-turn at 318 K though likely distorted is still present, differently to the two β-strands, whose diagnostic NOEs are virtually absent. At the same time, the analysis of aromatic regions of HPLW indicate that, at 318 K, Trp5 and Trp13 proton resonances, whose chemical shifts are far from their random coil value (Table S1), are still connected by inter-residue NOEs, therefore indicating that this hydrophobic cluster could play an important role in initiating the folding pathway of HPLW. The final input file for the CYANA structure calculation software contained 110 meaningful distance constraints (45 intra-residue, 50 sequential and 15 medium- and long-range) and 81 angle restraints (Table 1) and allowed the obtainment of a well resolved set of twenty calculated HPLW structures (Fig. 4a). As expected, the overall number of the NOE-derived distance constraints, which were 191 at 298 K, is significantly reduced at 318 K and this is a clear structural indication of HPLW thermally induced unfolding. In particular, the HPLW structure indicates that the temperature increase produces a significant but not complete loss of the peptide secondary structure. A comparison of the Ramachandran plots shows that ϕ and ψ backbone angles of residues 3–6 and 11–14 lie within the ranges typical of regular β-strands at 298 K but reside out of secondary structure typical ranges at 318 K (Figure S2). Accordingly, HPLW inter-strand hydrogen bonds (Tables S2 and S3) are not formed at 318 K. Differently, ψ and ϕ backbone angles of the residues 8 and 9 indicate the presence of a distorted of type II’ β-turn at 318 K, confirmed by the formation of a hydrogen bond connecting Asp10 HN and Arg7 CO (Fig. 4b).

**Figure 5:**
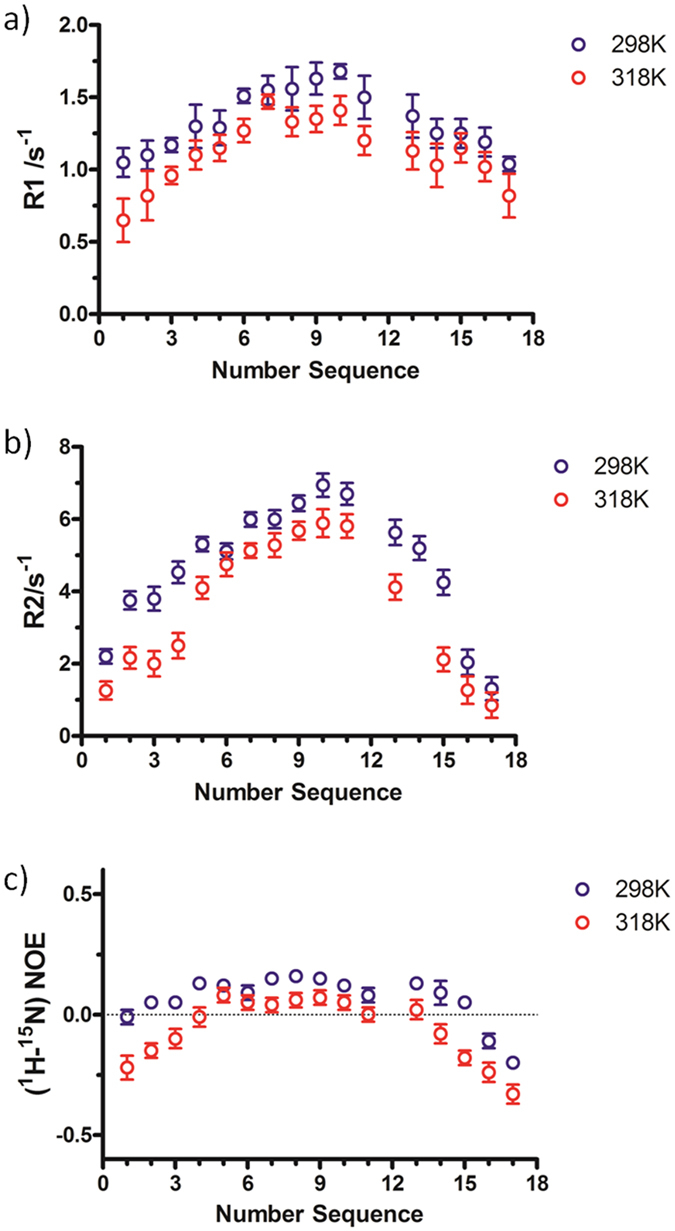
Experimental values of (**a**) ^15^N R1, (**b**) ^15^N R2 and (**c**) ^15^ N-{^1^H}-NOE as a function of residue number of HPLW measured in aqueous solution at 298 K and 318 K at 14.1 T.

^15^N-relaxation measurements have been carried out to provide information about internal and overall dynamics of the peptide at 298 and 318 K (Figure S3). In general, the trend in the relaxation rates is consistent with the decrease of backbone rigidity upon the temperature increase (Fig. 5). Particularly, reduction of R1 values appears constant along the entire peptide chain, whereas R2 decreases are more consistent in the β-strand forming residues^15^. N-{^1^H}-NOEs are more markedly reduced in the N- and C- termini and less decreased along the β-turn.

### Computational analysis

**Figure 6:**
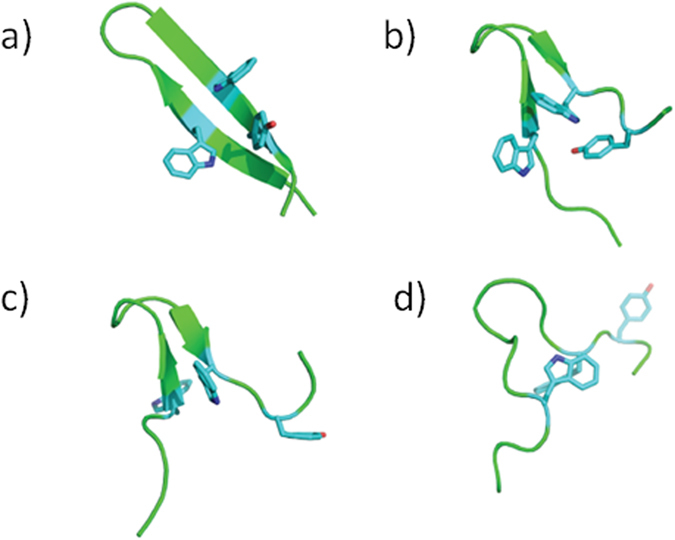
Representative structures from MD simulations at different temperatures. The figure report the structure of the centroid of the most populated clusters obtained from MD simulations at different temperatures. (**a**) 300 K; (**b**) 320 K; (**c**) 340 K; (**d**) 360 K. The side chains of the amino acids defining the hydrophobic contacts most relevant for the folding of the peptide are highlighted in light blue. The secondary structure elements were defined using the programs STRIDE^74^ and DSSP^75^.

To gain further insights into the mechanisms of HPLW stabilization and folding/unfolding, we performed different repetitions of MD simulations of the peptide at different temperatures (300, 320, 340, 360 K), covering a total time of about 3 microseconds. Analysis of time dependent backbone RMSD (Figure S4A and S5) from the native β-hairpin structure indicates that at 300 K the peptide mainly populates the native ensemble, with a well-conserved and stable amount of beta-structure. At 320K, the peptide visits a diverse range of conformational states. The analysis of relevant hydrogen-bonding interactions, as indicated by NMR analysis, confirms that the H-bond between Arg7 and Asp10 is consistently formed both at 320 and at 300 K (Figure S5). The further temperature increase to 340 and 360 K translates into peptide unfolding and sampling a wide range of different conformations. Structural clustering analysis (using the RMSD similarity criterion as in Daura *et al.*^47^) provides a structural view of the different ensembles: at 300 K the main cluster shows an ideal β-hairpin structure, with hydrogen bond pairing of facing residues along the strands, stabilized by a strong aromatic packing involving residues Trp5, Trp13 and Tyr15 (Fig. 6a). At 320 K, pronouncedly distorted structures appear, consistent with the population of a higher number of clusters. The most representative structures (still showing a degree of native character) are stabilized by packing of the aforementioned aromatic rings, with a distorted turn region (Fig. 6b). At 340 K, the structures of clusters 1, 3, 5, and 9 still represent the hairpin in the native structure with correct packing and turn geometry (Fig. 6c). These clusters account for 60%, 3%, 2% and 1%, respectively, of all sampled structures respectively, suggesting that the statistical frequency with which the native structure is populated is still significant even at this temperature and corroborating experimental observations on the peculiar persistency of beta-hairpin structures. Most of the remaining clusters (more than 60), however, indicate large conformational changes that take the peptide far from the native basin. At 360 K, the situation typical of random coils is observed (Fig. 6d). Consistent with the above reported observations, the flexibility (calculated in terms of residue-based Root Mean Square Fluctuations) of the peptide is very similar between 300 and 320 K, while a well-defined increase as a function of increasing temperature is observed for 340 and 360 K (Figure S6).

We next studied refolding (Figure S4B), starting from a completely extended peptide conformation and using plain MD simulations at 320, 340 and 360 K. At the two lower temperatures the peptide gets stuck in compact, partially ordered states. Three different simulations at 360 K were then run and analyzed. Interestingly, in the simulation labeled 360-1, the peptide spontaneously refolds to the native conformation (Figure S7). In this case, we observe the charged group of the arginine residues of the loop establishing charge-aromatic interactions with the tryptophan residues in the aromatic cluster. Arginine-aromatic interactions are always observed in alternative conformations, in particularly in out of register hairpins. Simulation 360-2 samples mainly unfolded conformations with the native one sporadically populated. In simulation 360-3, native-like packing is observed in β-hairpin conformations: the representative structures of clusters 1 pictorially depict this situation (Figure S8). In other hairpin-like structures (17 and 18) we observe that the partial formation of the aromatic cluster parallels the formation of hairpin-like structures; in many cases, the correct formation of aromatic core is accompanied by the formation of a distorted turn (Figure S7). Overall, these data provide a picture that is in qualitative agreement with the NMR experimental observations. In this context, they support a folding mechanism that entails the formation of a well-packed aromatic hydrophobic core, which drives the successive folding events.

### Design and stability analysis of HPLW mutants

Overall, the analysis of the thermally induced peptide folding/unfolding indicates that Trp5 and Trp13 interaction play a relevant role in nucleating and stabilizing HPLW β-hairpin conformation. To investigate the contribution of inter-strand Trp interactions to β-hairpin stability, two novel peptides were synthesized where Trp5 and Trp13 are replaced by a valine and named [5-Val]HPLW and [13-Val]HPLW, respectively. Valine has an intrinsic propensity towards β-conformations comparable to tryptophan, but nonetheless, is reported to form in β-hairpin weaker long range hydrophobic interactions^32^,^48^.

**Figure 7:**
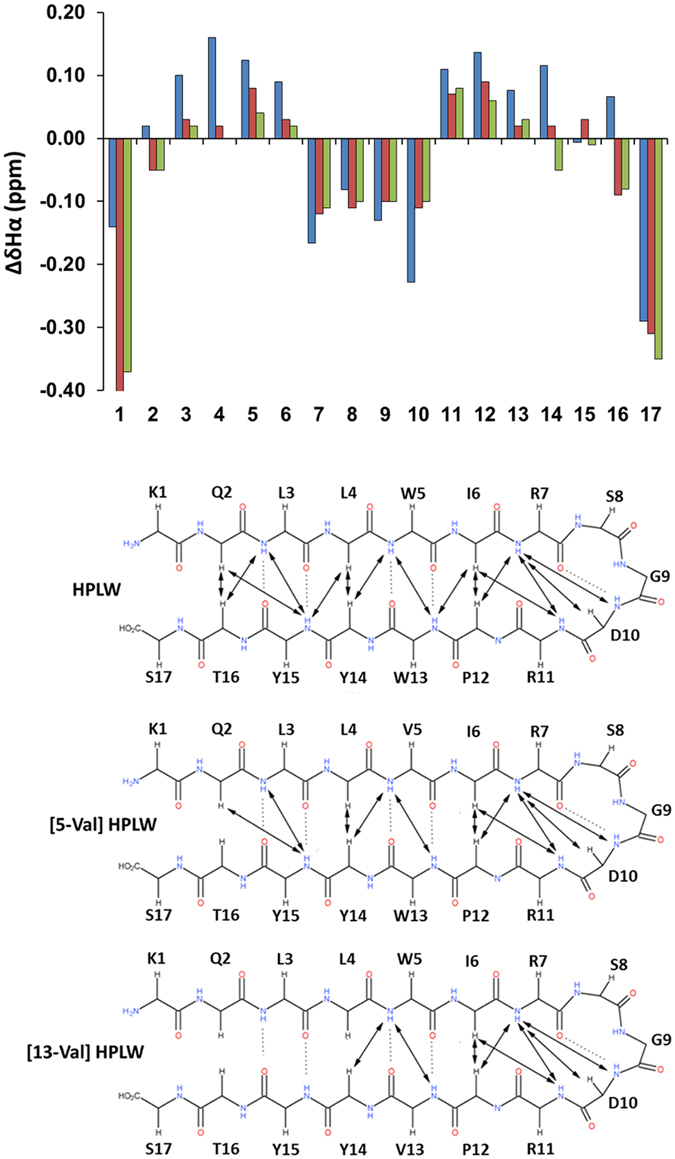
Upper: Deviation of Hα chemical shifts from random-coil values (ΔδHα) for HPLW (blue), [5-Val]HPLW (red) and [13-Val]HPLW (green) at pH 6.8 and 298 K. Lower: Schematic representation of β-hairpin peptide HPLW, [5-Val]HPLW and [13-Val]HPLW summarizing medium- and long-range NOEs.

The plot of ΔδHα values (Fig. 7 upper panel) for both hairpins indicate that these two mutations have a dramatic effect on the peptide conformations in water. Indeed, [5-Val]HPLW and [13-Val]HPLW do not contain stable β-strands, though preserving a turn conformation. Accordingly, the NOESY assignment shows a reduced number of NOE cross-peaks (Fig. 7 lower panel) with most of NOEs localized in the turn region. We have also characterized the thermal folding/unfolding pathway of the two peptides through NMR analysis. Interestingly, both [5-Val]HPLW and [13-Val]HPLW protons are all comprised in a single unfolding event centered at 311 ± 0.9 K (Figure S9).

## Discussion

HPLW is a designed VEGF receptor-binding peptide reproducing the PlGF region that is involved in receptor recognition. The NMR characterization has indicated that in aqueous solution the isolated peptide assumes a well folded β-hairpin conformation very similar to that observed in the context of the cognate protein. NMR interaction analyses revealed that the peptide binds to VEGFR1D2, both *in vitro* and in the cellular environment, and highlighted at the molecular level the residues involved in the interaction^41^. Biological characterization showed that HPLW has an *in vitro* VEGF-like activity, and, remarkably, is able to induce angiogenesis *in vivo*^37^. HPLW is one of the few pro-angiogenic peptides and its relevant biological activity is due to the β-hairpin conformation that allows the interacting residues to contact the receptor in the same three-dimensional arrangement as the natural ligand. We therefore undertook an accurate high-resolution characterization of the molecular mechanism driving the folding of HPLW β-hairpin structure. We have first determined the calorimetric parameters of the peptide thermal unfolding. DSC analysis clearly shows that HPLW unfolds reversibly and via a complex mechanism. In particular, the unfolding occurs in a rather large temperature range of about 50 K, that is centered around 316 K. ΔH^VH^ is quite large for a 17-mer peptide, in agreement with previous findings^24^. Importantly, r^VH^ is significantly higher than 1, indicating that the unfolding process does not proceed through a typical cooperative mechanism, but it should consist of a more complex sequence of folding events. The NMR analysis has allowed following the single unfolding process of many of the ^1^H nuclei comprised in HPLW peptide. A first inspection of the Hα behavior in 283–343 K range (Fig. 2 and S1) indicates that the peptide starts to lose secondary structure content at around 300 K and completes its unfolding process just before 340 K. More in detail, proton melting temperatures can be grouped in three main clusters of residues, exhibiting Gaussian-type distributions of about 2 K around the central values (Fig. 3). In particular, Trp5 and Trp13, which lie at the edges of the two β-strands, fold at 320.1 K almost concurrently with all the β-turn forming residues that fold at 319.3 K. Differently, residues composing the rest of the β-strands fold at 316.6 K, concluding the peptide secondary structure formation. To confirm this sequence of folding events, we carried out the structural analysis, by means of NMR techniques, of HPLW at 318 K. The comparison of the HPLW solution structures derived at 298 and 318 K (Fig. 4b,c) clearly shows that upon 20 K of temperature increase the peptide still preserves a bent conformation, stabilized by a distorted type II β-turn and by a long range Trp5-Trp13 aromatic ring interaction, nonetheless losing the anti-parallel β-sheet structure. Upon this reduction of secondary structure the N- and C-terminal ends of HPLW come sensibly farer apart, at 318 K, than they occur at 298 K (Fig. 4). Accordingly, the flexibility of the backbone peptide chain, as derived by ^15^N relaxation measurements, increases more significantly, particularly in the range of the micro- to milli-second motions, within the β-strands and at the N- and the C-termini (Fig. 5).

The analysis of the melting temperatures of single protons together with the structural preferences of HPLW at 318 K and accordingly with DSC results accurately outline the peptide folding mechanism, in agreement with the computational folding/unfolding simulations. The HPLW folding process consists of two main events, which are successive but do not superimpose. The first folding step initiates at 320 K upon the hydrophobic collapse of the Trp5 and Trp13 side-chains which stabilizes the concurrent β-turn formation, whose CO_i_-HN_i + 3_ hydrogen bond (Asp10 → Arg7) appears particularly stable, as indicated either by the experimental and computational analysis (Figs 4 and 6).

This initial folded structure is also strengthened by non-native cation-π interactions forming between Arg7 and Arg13 side chains with Trp 5 and Trp13 indolic rings, respectively, well described both by the NMR structure and by the refolding simulations (Figure S7 and S10). At 316 K, once the β-turn is completely formed, the two β-strands pair, very likely starting, as indicated by the NMR and computational analyses, by Trp5 and Trp13, which thus play a key role also in the final step of the β-hairpin folding. The picture emerged from MD-based studies confirm the fundamental role assumed by the long-range Trp aromatic interaction in the β-hairpin formation. To further corroborate this evidence, we designed two single HPLW mutants, [5-Val]HPLW and [13-Val]HPLW, in which each of the two Trp residues are replaced by a valine.

The conformational preferences of the two mutants show the loss of the β-hairpin structure in both the peptides, which nonetheless preserves a bent conformation (Fig. 7). Remarkably, in both the peptides the turn structure unfolds in a single event centered around 311 K (Figure S9). These results clearly indicate that the long-range hydrophobic interaction forming at the beginning of the β-strands is crucial to stabilize the β-turn formation and to start the β-strand pairing. When the strength of this interaction is reduced, upon replacement of one Trp with a Val^32^,^48^, the first folding event occurs at a 9 K lowest temperature and the second folding event cannot occur.

Marginal stability, long folding times compared to helices and strong tendency to aggregate have made β-hairpin forming peptides in general difficult to study^49^. Nonetheless, they represent an essential tool to investigate the initial steps of protein folding. In the last two decades a growing number of short peptide sequences, even smaller than 10 residues, have been designed and demonstrated to adopt monomeric, water soluble β-hairpin conformations up to 95% of their population^33^,^50^,^51^. These peptides have been used as models for studying secondary structure propensities free of the context-dependent tertiary interactions found in protein β-sheets. To this aim, folding pathways of β-hairpin forming peptides have been characterized, making use of different experimental, mainly NMR and DSC, techniques and of computational analysis^5^,^24^,^28^,^29^,^30^,^31^,^32^. In this study we have exploited a multi-technique approach, combining DSC, NMR, MD analyses and the design of mutants, that aims to provide a detailed picture of the folding pathway of a designed highly structured β-hairpin forming peptide. In particular, a thorough analysis of single proton chemical shift perturbations upon thermal unfolding provides a very useful tool to outline the high-resolution HPLW folding pathway, which, as also confirmed by the DSC and computational analysis is not composed of a single cooperative event. Previously, chemical shifts perturbations to monitor thermal unfolding pathways of model peptides had been reported mainly utilizing only few specific well-behaving protons^52^,^53^. Our results indicate that HPLW folding pathway does not show evidence of cooperativity, typical of globular proteins; in contrast, the formation of the HPLW β-hairpin entails two successive steps, triggered by the propensity of HPLW sequence to form a turn which is strongly stabilized by aromatic packing. This non cooperative and fast secondary structure formation can be recapitulated by the concept of formation of Local Elementary Structures (LES)^54^. Such simple sub-structures can initiate full protein folding by forming locally ordered structures due to the physical proximity of the aminoacids in the sequence. Once a minimal number of LES is formed in the protein sequence, these can aptly trigger a more complex cooperative folding events typical of larger proteins.

Recently, accurate structural studies of G-hairpin peptide performed at different temperatures revealed that two factors are essential for formation-stabilization of structure in the β−hairpin forming peptides, i.e. hydrophobic interactions between non polar side chains and turn formation propensities of part of the sequence^29^,^55^,^56^,^57^. Accordingly, our experimental and computational analysis, point out that HPLW β-hairpin formation requires the long range Trp5-Trp13 interaction and the concurrent stabilization of β-turn which is crucial to allow the β-sheet stabilization at room temperature. Indeed, upon the substitution of a single Trp with a Val, the peptide chain is able to form a turned structure but not to fold stably as a β-hairpin.

If one wants to define the pathway of HPLW β-hairpin formation following the classification proposed by Scheraga and coworkers^32^, the zipper, the hydrophobic collapse and the broken-zipper mechanisms, this falls in the latter category. The HPLW folding cannot be described as based on zipper mechanism, since hydrogen-bonding formation does not propagate driving the peptide folding; neither by the hydrophobic collapse mechanism, which ignores the importance of the turn-region structure in the β-hairpin formation, Indeed, in HPLW folding mechanism, long range hydrophobic interactions coupled with a certain intrinsic propensity to form the turn region plays a key role in initiating the β-hairpin formation, two factors that characterize the broken-zipper mechanism. In this mechanism, at the early stage of the folding the turn and the contacts are not required to be very stable, but, importantly, they already lie in about the correct final sites, providing sufficient robustness and uniqueness to the folding pathway.

Overall, here we describe a multi-state hierarchical folding pathway of a highly structured β-hairpin, which consists of two successive main folding events and can be classified as a broken-zipper mechanism. Importantly, similar mechanisms of β-hairpin formation could play fundamental roles in starting the folding of larger sequence native structures at the same time hampering the trigger of intermolecular interactions during the physiological life of the protein.

## Methods

### Peptide synthesis

Synthesis and characterization of the HPLW peptide (NH2-KQLLWIRSGDRPWYYTS-OH) has been previously described^37^. Mutant peptides [5-Val]HPLW (NH2-KQLLVIRSGDRPWYYTS-OH) and [13-Val]HPLW (NH2-KQLLWIRSGDRPVYYTS-OH) were synthesized by solid-phase peptide synthesis using Fmoc chemistry. The syntheses were carried out on the NovaSyn TGA (90 μm) resin (Merk Millipore, Vimodrone (MI), Italy) (loading 0.24 mmol g-1) using Fmoc-amino acids with standard side-chain protecting groups (Iris-Biotech, Marktredwitz, Germany). The first amino acid was loaded using 10 eq of the Fmoc-amino acid dissolved in dichloromethane (DCM) (Sigma-Aldrich, Milan, Italy) and preactivated with 5 eq of N,N′-diisopropylcarbodiimide (DIC) (Sigma-Aldrich, Milan, Italy) for 20 min on ice with stirring. Then DCM was removed under nitrogen flux, and the mixture was dissolved in N,N-dimethylformamide (DMF) (Romil, Cambridge, UK). The solution was added to the resin with 0.1 eq of 4-dimethylaminopyridine (DMAP) (Sigma-Aldrich, Milan, Italy). The reaction was carried out for 2 h at room temperature. The procedure was repeated twice. The removal of Fmoc was carried out by incubating the resin with a solution of 20% v/v piperidine (Biosolve, Valkenswaard, The Netherlands) in DMF twice for 7 minutes. Each coupling reaction was performed for 30 min using 5 equivalents of the Fmoc-protected amino acid, 4.99 equivalents of 1 [Bis(dimethylamino)methylene]-1H-1,2,3-triazolo[4,5-b]pyridinium 3-oxid hexafluorophosphate (HATU) (GL Biochem, Shangai, China) and 10 equivalents of the base N,N-diisopropylethylamine (DIPEA) (Sigma-Aldrich, Milan, Italy). After each coupling reaction, unreacted N-terminal amino groups were capped with a solution of 2 M acetic anhydride (Sigma-Aldrich, Milan, Italy), 0.55 M DIPEA in 1-methyl-2-pyrrolidinone (NMP) (Romil, Cambridge, UK) (5 min). Each reaction step was followed by five washing with DMF for 1 min. Peptides cleavage from the resin and amino acid side chains deprotection were achieved by treatment with trifluoroacetic acid (TFA) (Sigma-Aldrich, Milan, Italy), triisopropylsilane (TIS) (Sigma-Aldrich, Milan, Italy) and water (95 : 2.5 : 2.5) at room temperature for 3 h. Cold diethyl ether was used to precipitate the peptides. Crude products were collected by centrifugation, resuspended in a water–acetonitrile mixture and lyophilized. The peptide were purified by reverse-phase HPLC on a HP 1200 Series (Agilent Technologies) using a AXIA RP-MAX Synergi column (4 μ, 80 Å, 50 × 21.2 mm; Phenomenex, Torrance, US) applying a linear gradient of CH_3_CN (0.1% TFA) in H_2_O (0.1% TFA) from 20% to 50% in 20 min using a flow rate of 20 mL/min. Pure peptides were finally lyophilized. Peptides purity (>95% based on the analytical HPLC area revealed at 210 nm) and identity were verified by LC-MS on an Agilent 1200 Infinity Series (Agilent Technologies, Santa Clara, CA, US) chromatographic system equipped with a diode array combined with an electrospray ion source and a time-of-flight mass analyzer using a C18 Jupiter column 150 × 2 mm, 300 Å, 3 μm (Phenomenex, Torrance, US) and applying a gradient of CH_3_CN (0.1% TFA) in H_2_O (0.1% TFA) from 20 to 70% in 20 min at a flow rate of 0.2 ml/min.

[5-Val]HPLW: MW_calc_: 2081.1057 Da; MW_exp_: 2081.1067 Da. t_R_ 9.74 min.

[13-Val]HPLW: MW_calc_: 2081.1057 Da; MW_exp_: 2081.1072 Da. t_R_ 9.16 min.

Preparation of ^15^N-HPLW. ^15^N labelled HPLW peptide was prepared by recombinant expression in *E. coli* fused to MxeGyrAintein as previously reported^41^.

### DSC analysis

DSC experiments were carried out with a MicroCal VP-DSC calorimeter. All peptide samples were dissolved in neutral ultrapure MilliQ water and, after a degassing process, were heated at 1 K/min in the temperature range 280–350 K. An extra external pressure of about 29 psi was applied to the solution to prevent the formation of air bubbles during heating. In all measurements, ultrapure MilliQ water was used in the reference cell of the calorimeter. In order to ensure a proper equilibration of the calorimeter, several water-water heating scans were routinely performed prior to the measurement. Only after obtaining invariant water-water baselines scans of HPLW (870 μM) were performed. Further baselines were obtained immediately after the peptide scans to rule out uncontrolled drifts in instrumental baseline. To obtain the heat capacity Cp curves, buffer–buffer base lines were recorded at the same scanning rate and then subtracted from sample curves, as previously reported^58^,^59^,^60^. In most experiments, one or several heating-cooling cycles were carried out to determine the reversibility of the denaturation process. In the case of HPLW, as for most of very small proteins or peptides^61^, the native baseline cannot be directly extrapolated from the DSC thermogram. As an alternative to direct extrapolation, to obtain the excess heat capacity profiles (Cp_exc_) of HPLW the raw calorimetric data, after instrumental baseline correction, were subtracted from a baseline obtained by a linear interpolations of the onset and offset transition points as shown in Fig. 1.

### Nuclear Magnetic Resonance Spectroscopy

For HPLW thermal unfolding experiments the NMR sample was prepared by dissolving the lyophilized peptide at a concentration of 1.0 mM in in 90% H_2_O and 10% ^2^H_2_O mixture at pH 6.8. All of the NMR spectra were carried out on a Varian Inova 400 MHz spectrometer, where the probe temperature was regularly calibrated by using methanol and ethylenglycol^62^. For the thermal unfolding experiments, a series of homonuclear 2D-TOCSY spectra were acquired increasing temperatures at regular intervals of 5 K from 278 to 343 K, recording all the spectra consecutively. HPLW unfolding curves were obtained by fitting with GraphPad Prism5 software [ www.graphpad.com]. Particularly, a single step function resulted in stable fits with no systematic deviations from the experimental curve (Equation 2), in which *A1* and *A2* are the starting and the final amplitudes, *B* is the slope of the baseline, *x*_*0*_ is the midpoint or transition point, and *dx* is the slope at *x*_*0*_:




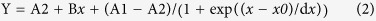




For HPLW solution structure at 318 K NMR spectra were acquired on a Varian Inova 600 MHz spectrometer (*Varian Inc., Palo Alto, CA, USA*), equipped with a cryogenic probe optimized for ^1^H detection. Different mixing times were used to evaluate the linear build-up of NOE and to find the mixing time appropriate at 318 K; NOESY spectrum recorded with a mixing time of 350 ms was chosen for obtaining the distance constraints. Double quantum filtered spectroscopy (DQF-COSY) was recorded with 4096 data points in the direct dimension and with 500 increments each comprising 64 scans to obtain enough resolution to measure the ^3^J_HNHα_ coupling constants. Water suppression was achieved by means of Double Pulsed Field Gradient Spin Echo (DPFGSE) sequence^63^,^64^.

All spectra were processed with the software Sparky^65^ and analyzed with Neasy, a tool of CARA software^66^. Experimental distance restraints for structure calculations were derived from the cross-peak intensities in NOESY spectra. Distance constraints together with 10 coupling constants were then used by the GRIDSEARCH module, implemented in CYANA software^67^, to generate a set of allowable dihedral angles. Structure calculations, which used the torsion angle dynamics protocol of CYANA, were then started from 100 randomized conformers. The 20 conformers with the lowest CYANA target function were further refined by means of restrained energy minimization, using the Gromos 96 force field, with the program SPDB VIEWER^68^ The color figures and the structure analysis have been performed with the program MOLMOL^69^.

The ^15^N relaxation parameters^70^ R1, R2 and ^15^N-{^1^H}-NOE were determined at 600 MHz at 298 K and 318 K. 2D [^1^H-^15^N] heteronuclear single quantum coherence (HSQC) correlation spectra for ^15^N uniformly labeled HPLW were acquired using a spectral width with 1024 (HN) × 128 (N) data points and 16 (for R1 and R2) or 64 (for NOE) scans. ^15^N R1 experiments were recorded with relaxation delays of 10, 50, 100, 150, 200, 300, 400 ms while ^15^N R2 experiments spectra were acquired with relaxation delays of 15, 30, 60, 90, 120, 150, 165 ms. Steady state {^1^H}-^15^N NOE spectra were recorded with and without a 3 s pre-saturation period. During this time, proton frequencies were irradiated with a continuous low-power pulse. In the experiment without pre-saturation, the low power irradiation was replaced with a delay period of 3 s. Peak intensities were measured for the calculation of the relaxation times and heteronuclear ^15^N-{^1^H}-NOE values. R1 and R2 rates were determined by fitting peak intensities (*I*) at multiple relaxation delays (*t*) to the equation *I*_*(t)*_ = *I*_*0*_*e*^*−Rit*^. Uncertainties in R1 and R2 were obtained from the statistical errors obtained by fitting. ^15^N-{^1^H}- steady-state NOEs were calculated as the ratio of ^1^H-^15^N correlation peak heights in the spectra acquired with and without proton saturation and their uncertainties were set to 5%.

[5-Val]HPLW and [13-Val]HPLW thermal unfolding experiments were performed under conditions identical to those used for HPLW described above.

### Computational analysis

All MD simulations were performed using the AMBER 12.0 package^71^ and force field, the TIP3P water model^72^, and the Particle Mesh Ewald summation method (PME) to deal with long-range Coulomb interactions^73^. Counterions were added randomly to ensure charge neutrality. The time step for the integration of the equations of motion was 0.002 ps. Systems starting from the native structure were simulated for 250 ns each. 4 simulations were sun at 300, 320, 340 and 360 K. Systems starting from the completely extended structure were simulated for 150 ns, at 320, 340 and 360 K. In the case of the highest temperature 3 copies were run, starting with different initial velocities. Frames for analysis were saved every 10 ps for each system. All structural analyses were carried out using cpptraj/ptraj module as implemented in Amber Tools 13^71^.

## Additional Information

**How to cite this article**: Diana, D. *et al.* Long range Trp-Trp interaction initiates the folding pathway of a pro-angiogenic β-hairpin peptide. *Sci. Rep.* **5**, 16651; doi: 10.1038/srep16651 (2015).

## Supplementary Material

Supplementary Information

## Figures and Tables

**Table 1:** Structural statistics of the 20 final NMR structure of the HPLW at 318 K.

Number of the distance restraints	
Unambiguous NOE	110
Ambiguous NOE	71
Total NOE	181
Divided into	
Intra-residue NOE	45
Sequential NOE	50
Medium, Long-range NOE	15
Number of dihedral angle restraints	81
Residual NOE violations	
Number > 0.1°	±1
Maximum, Å	0.18 ± 0.02
Residual angle violations	
Number > 2.0°	0 ± 0
Maximum, Å	0
Amber energies, KJ/mol	
Total	−410 ± 25
Van der Waals	−215 ± 20
Electrostatic	−282 ± 18
R.m.s.d (Å) to a mean structure	
Backbone (residues 5–13)	0.49 ± 0.05
Heavy atoms (residues 5–13)	1.33 ± 0.09
