# Supplementary material for: Long range Trp-Trp interaction initiates the folding pathway of a pro-angiogenic β-hairpin peptide
Source: Sci Rep. 2015 Nov 25;5:16651. doi: 10.1038/srep16651 (PMC4658480; doi:10.1038/srep16651)
Supplement: Supplementary Information [file srep16651-s1.doc]

**Supporting Information**

Long range Trp-Trp interaction initiates the folding pathway of a pro-angiogenic β-hairpin peptide

Donatella Diana, Lucia De Rosa, Maddalena Palmieri, Anna Russomanno, Luigi Russo, Carmelo La Rosa, Danilo Milardi, Giorgio Colombo, Luca D. D’Andrea and Roberto Fattorusso

**Contents:**

**Figure S1**: Representative melting curves of HPLW protons.

**Figure S2**: Ramachandran plots for HPLW at 298 K (red) and at 318 K (blue).

**Figure S3**: 2D [^15^N, ^1^H] HSQC relaxation spectra of ^15^N HPLW at 318 K and at 14.1 T with τ = 10 ms and τ = 400 ms for R1 measurements (A) and τ= 15 ms e τ = 165 ms for R2 measurements (B).

**Figure S4**: (A) Time evolution of the RMSD of the peptide backbone from the NMR-determined native structure of simulations started from the same native conformation. Different colors identify different simulations: Black, 300K; Red, 320K; Green, 340K; Blue, 360K. (B) Time evolution of the RMSD of the peptide backbone from the NMR-determined native structure, in the simulations started from a completely extended conformation. Different colors identify different simulations: Black, 320K; Red, 340K; Green, 360K run 1; Blue, 360K run 2.

**Figure S5:** Time evolution of the distances between selected side chains in the simulations started from the native structure.

**Figure S6**: Evaluation of peptide flexibility as a function of temperature. Root Mean Square Fluctuations (RMSF) were computed for the backbone atoms of each system after superimposing the backbone atoms of all structures in each trajectory onto the energy minimized, NMR-derived initial structure. Different colors identify different simulations: Black, 300K; Red, 320K; Green, 340K; Blue, 360K

**Figure S7**: Time evolution of the secondary structure contents in the 360 K refolding simulations started from the completely extended structure of the peptide.

**Figure S8**: Representative structures from refolding simulation.

**Figure S9**: Representative melting curves of (A) [5-Val]HPLW and (B) [13-Val]HPLW protons.

**Figure S10**: Superposed backbone traces for the NMR-derived structural ensemble of HPLW at 318 K. The side chain of Trp5, Arg7, Arg11 and Trp13 are represented as neon.

**Table S1**: ^1^H chemical shift assignment of the HPLW peptide at 318 K.

**Table S2**: Observed and calculated average hydrogen bond lengths for HPLW at 298K.

**Table S3**: Observed and calculated average hydrogen bond lengths for HPLW at 318K**.**

**
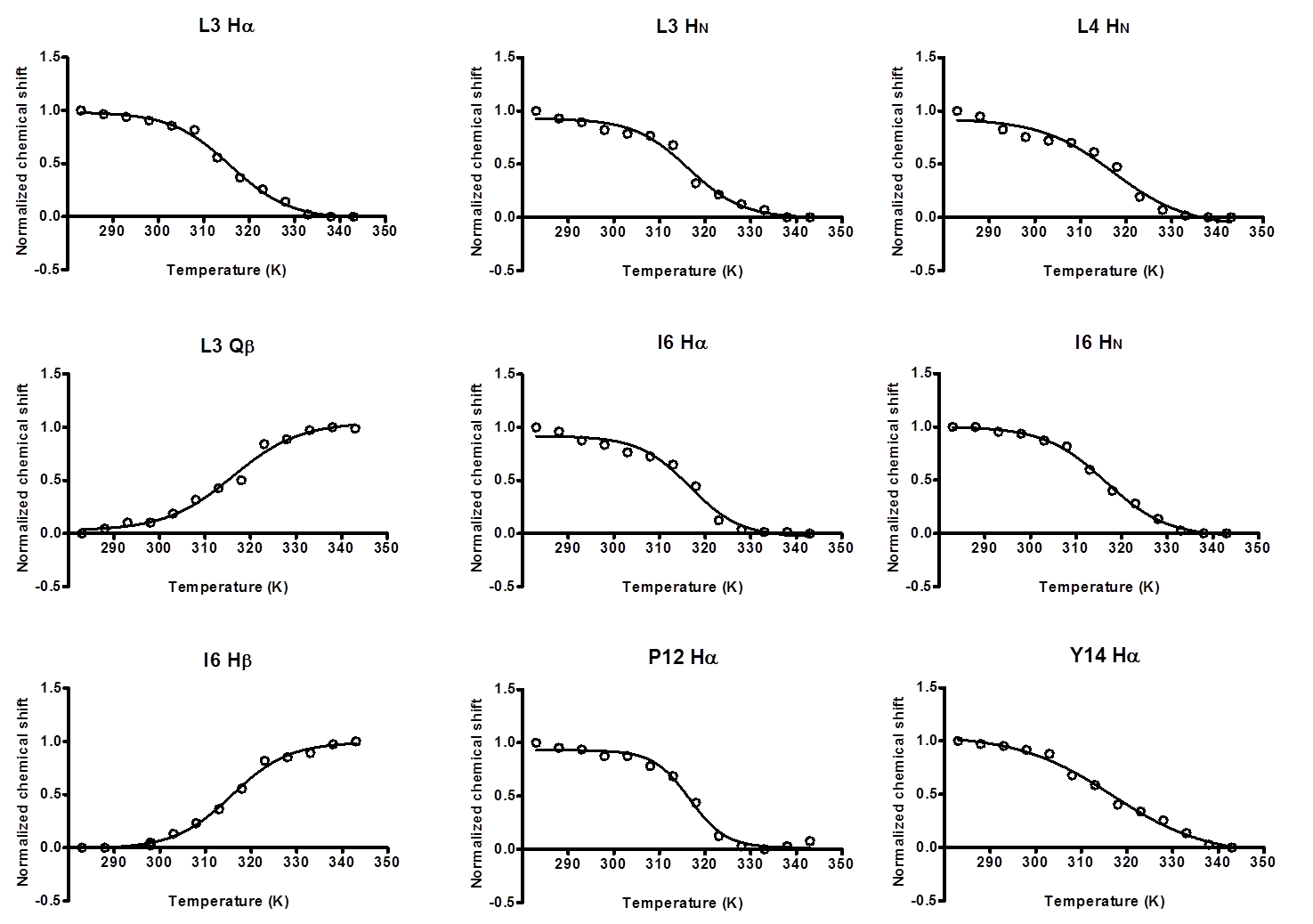
**


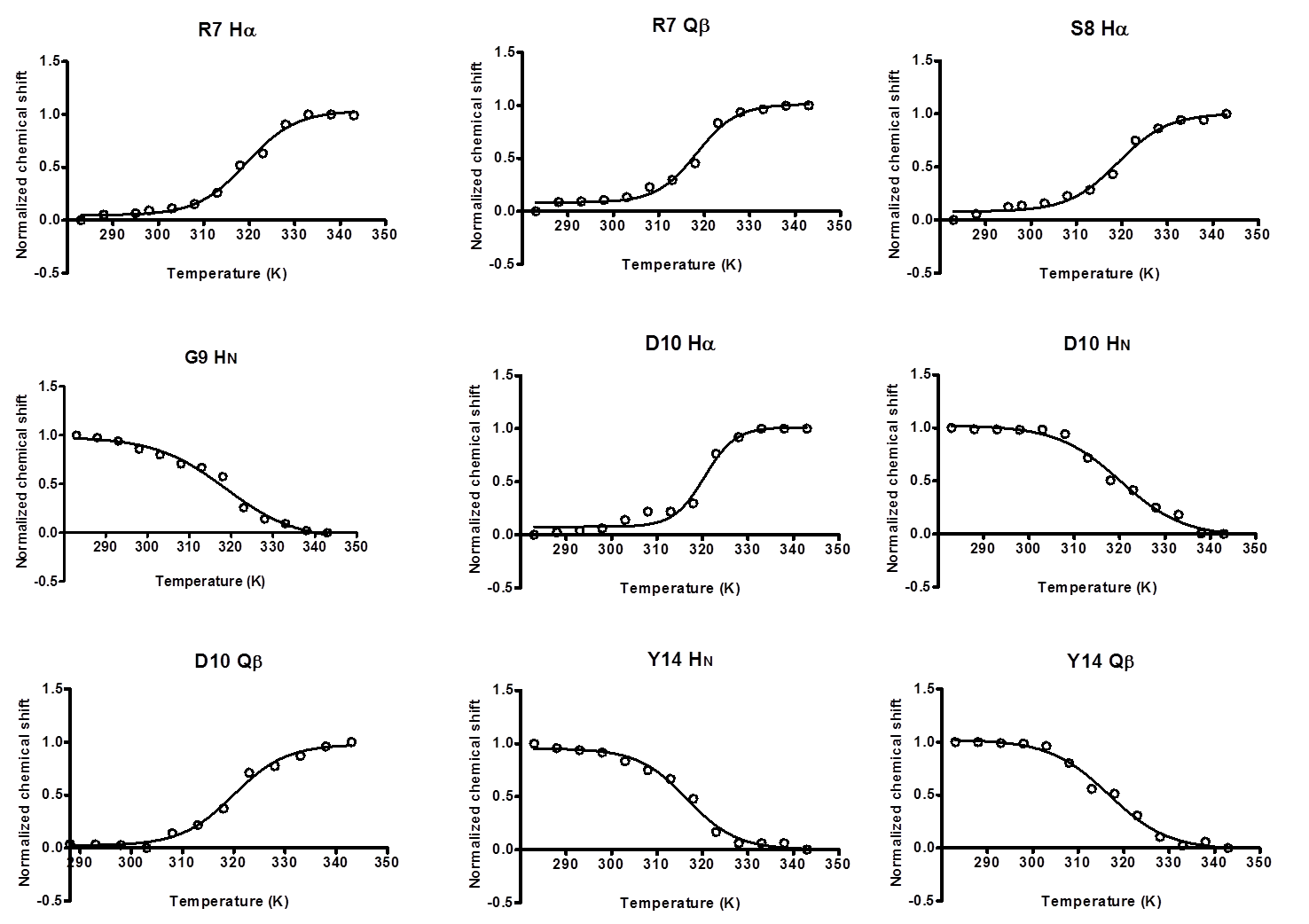


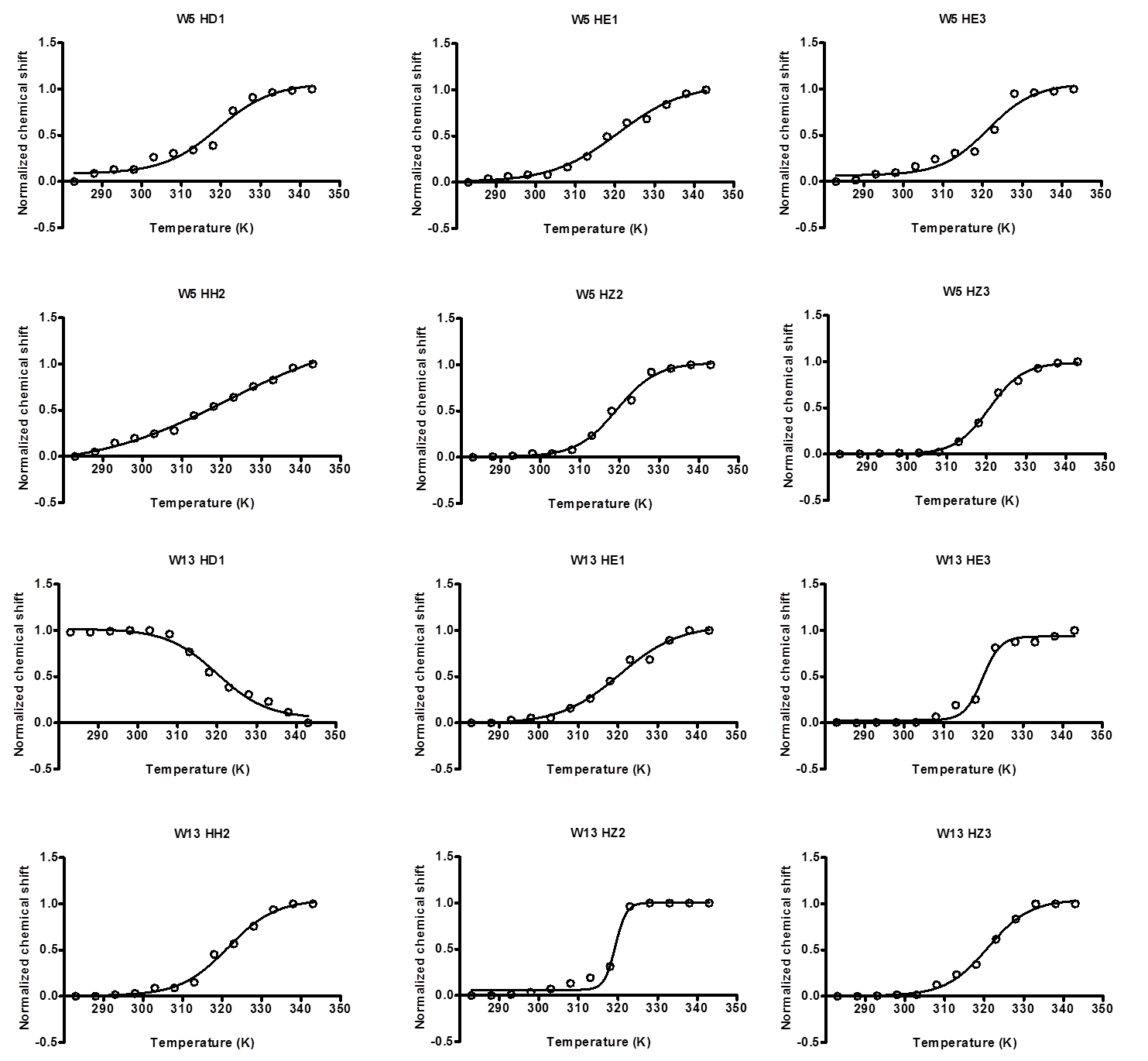


**Figure S1**: Representative melting curves of HPLW protons.


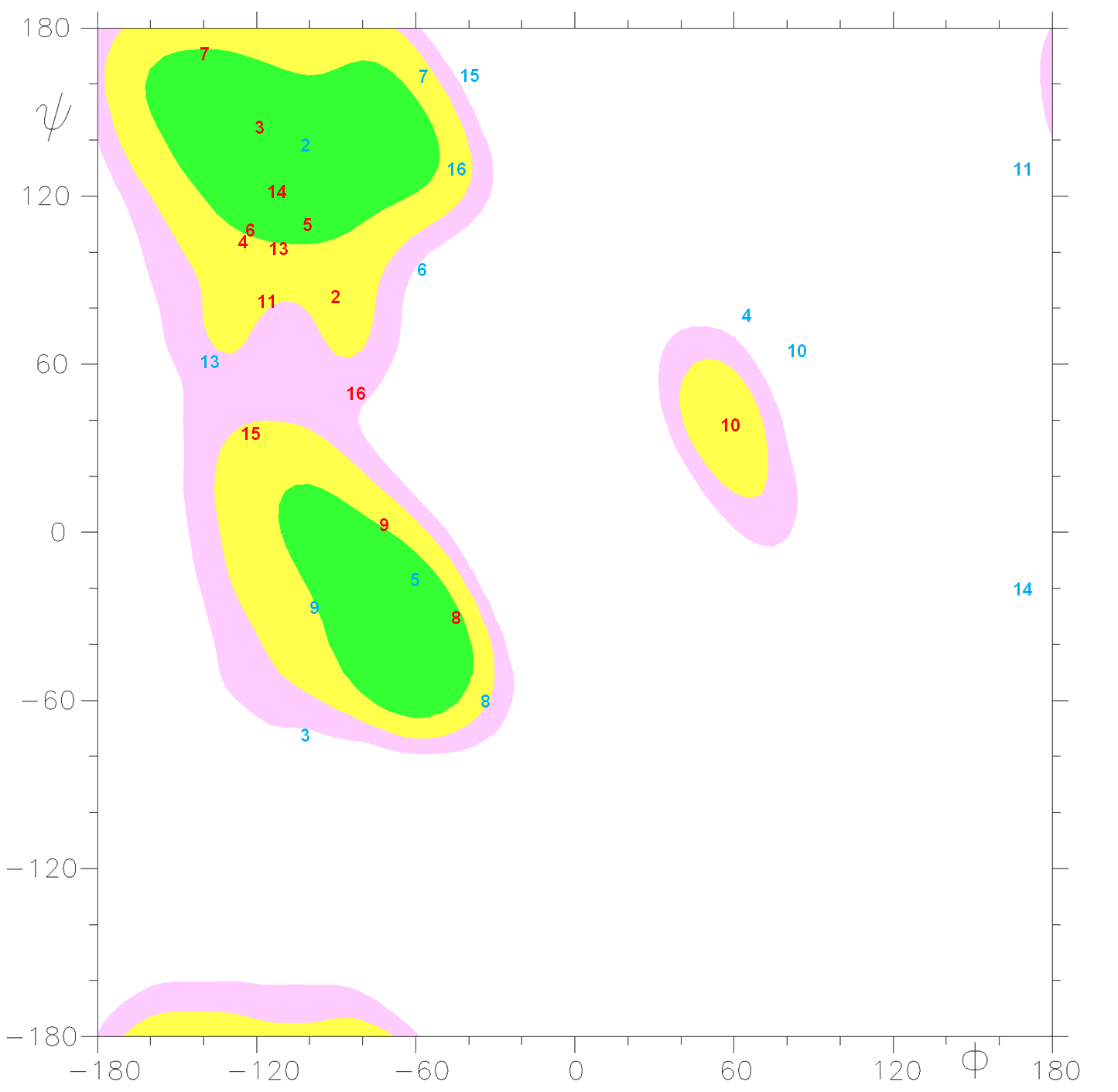


**Figure S2**: Ramachandran plots for HPLW at 298 K (red) and at 318 K (blue).


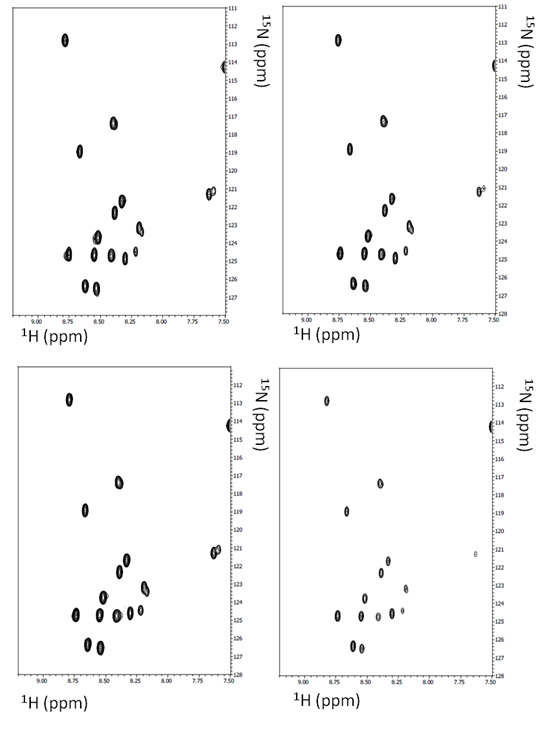


A)

B)

**Figure S3**: 2D [^15^N, ^1^H] HSQC relaxation spectra of ^15^N HPLW at 318 K and at 14.1 T with τ = 10 ms and τ = 400 ms for ^15^N R1 measurements (A) and τ= 15 ms e τ = 165 ms for ^15^N R2 measurements (B).

**
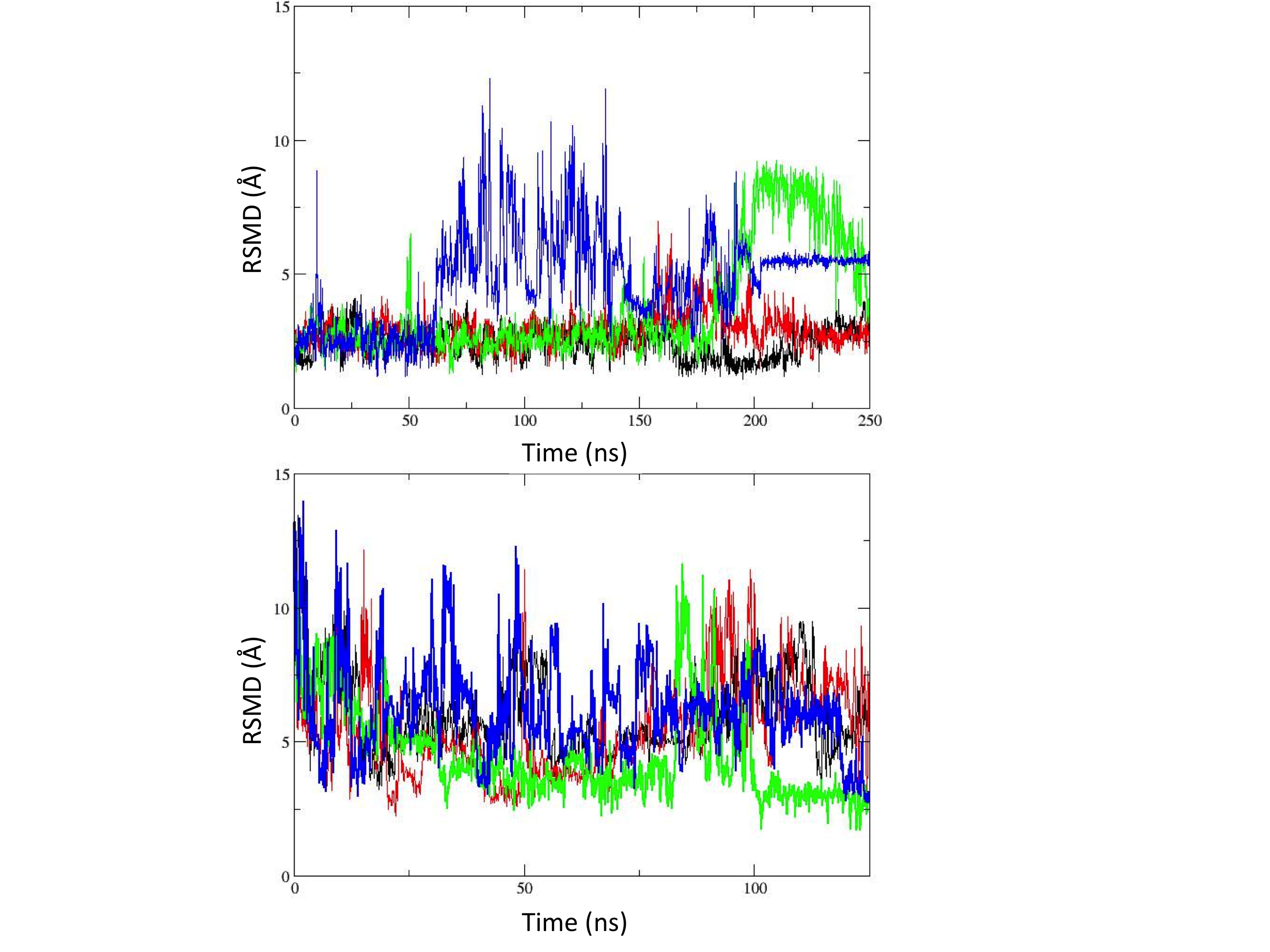
**

**Figure S4:** (A) Time evolution of the RMSD of the peptide backbone from the NMR-determined native structure of simulations started from the same native conformation. Different colors identify different simulations: Black, 300K; Red, 320K; Green, 340K; Blue, 360K. (B) Time evolution of the RMSD of the peptide backbone from the NMR-determined native structure, in the simulations started from a completely extended conformation. Different colors identify different simulations: Black, 320K; Red, 340K; Green, 360K run 1; Blue, 360K run 2.


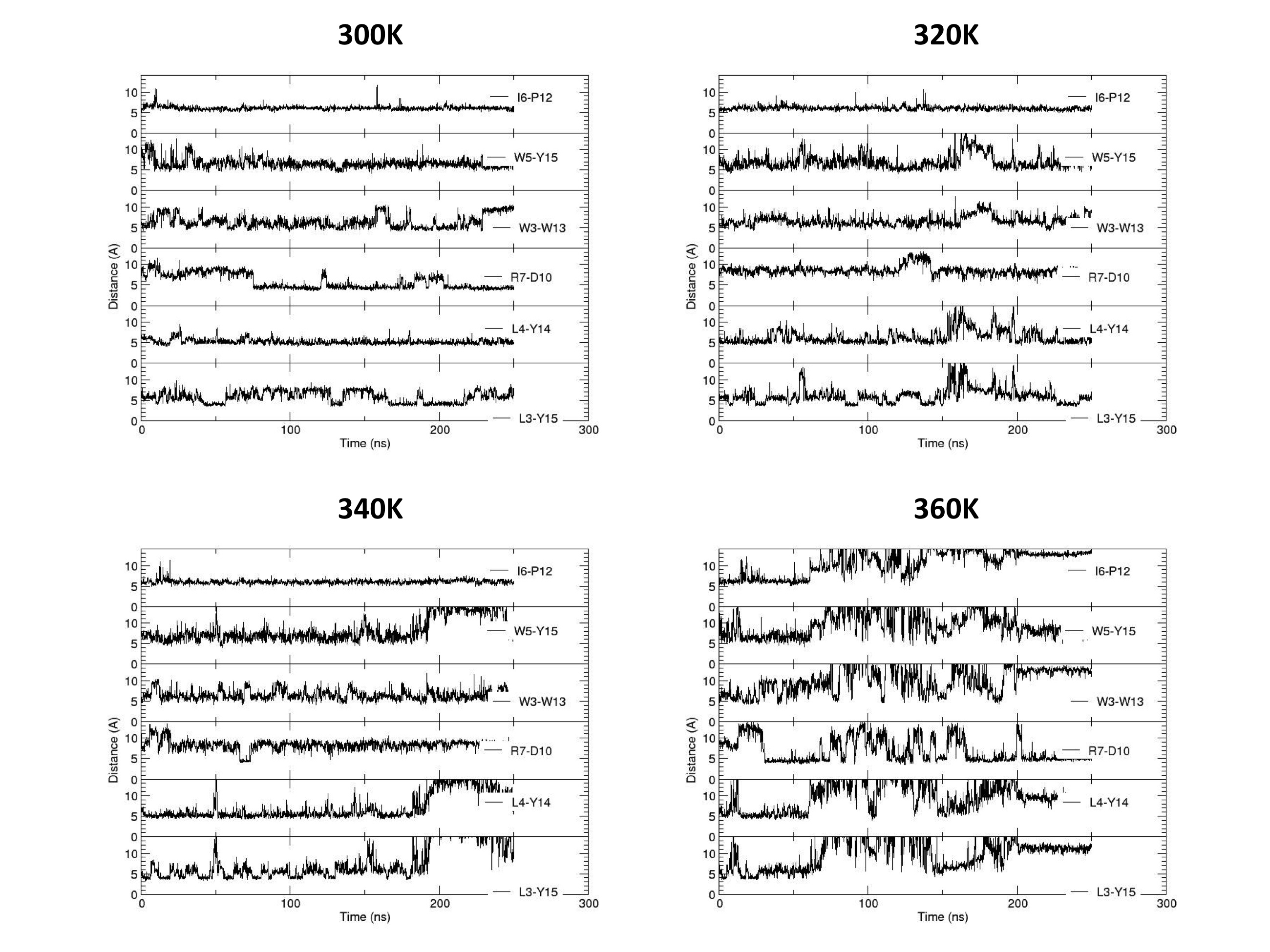


**Figure S5:** Time evolution of the distances between selected side chains, in the simulations started from the native structure. The distance between the amino acids is calculated as the distance between the centers of masses of the residues.


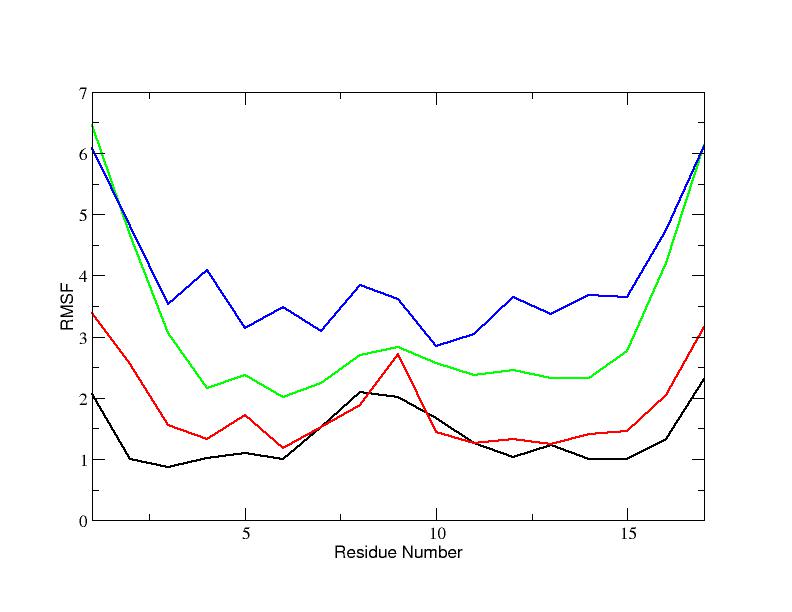


**Figure S6:** Evaluation of peptide flexibility as a function of temperature. Root mean square fluctuations (RMSF) were computed for the backbone atoms of each system after superimposing the backbone atoms of all structures in each trajectory onto the energy minimized, NMR-derived initial structure. Different colors identify different simulations: Black, 300K; Red, 320K; Green, 340K; Blue, 360K


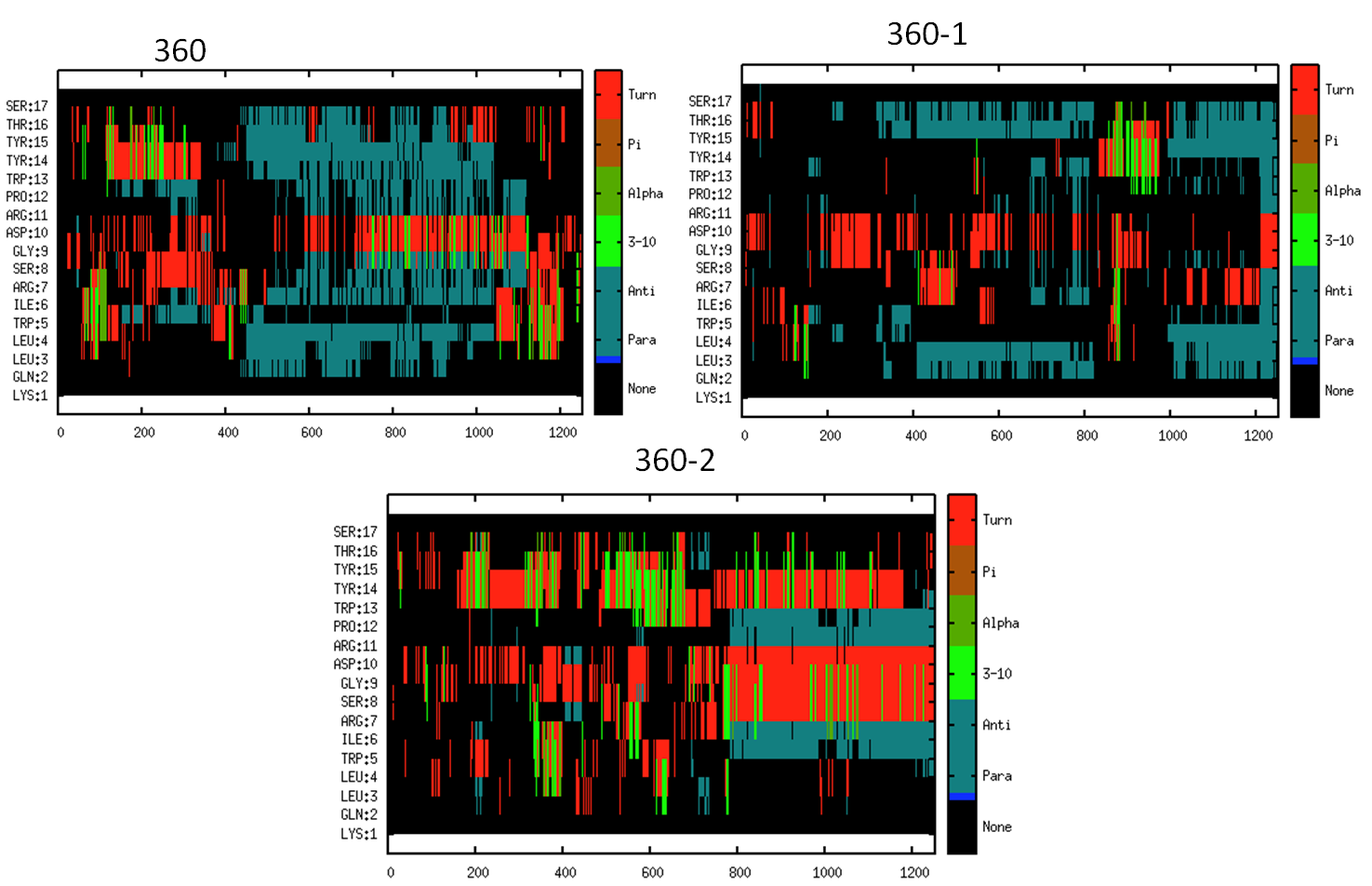


**Figure S7**: Time evolution of the secondary structure contents in the 360 K refolding simulations started from the completely extended structure of the peptide.


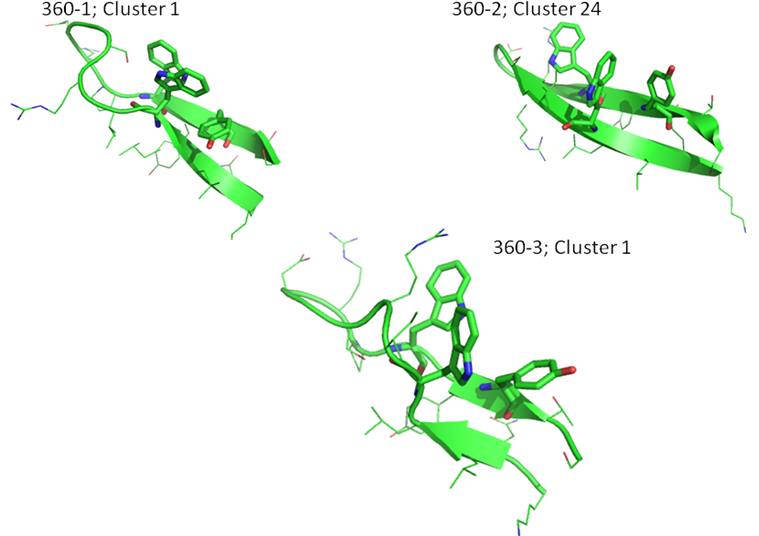


**Figure S8**: Representative structures from refolding simulation.


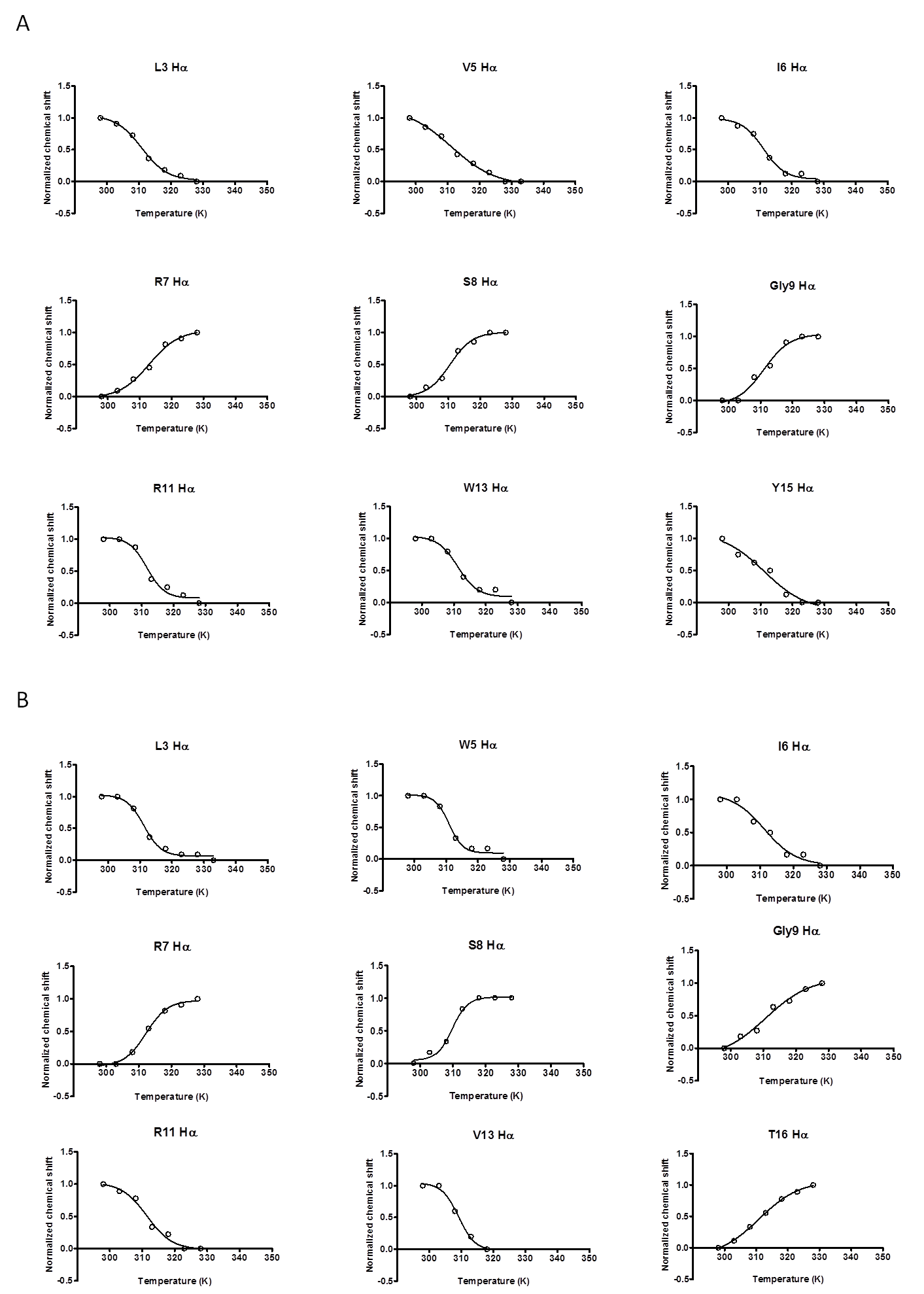


**Figure S9**: Representative melting curves of (A) [5-Val]HPLW and (B) [13-Val]HPLW protons.


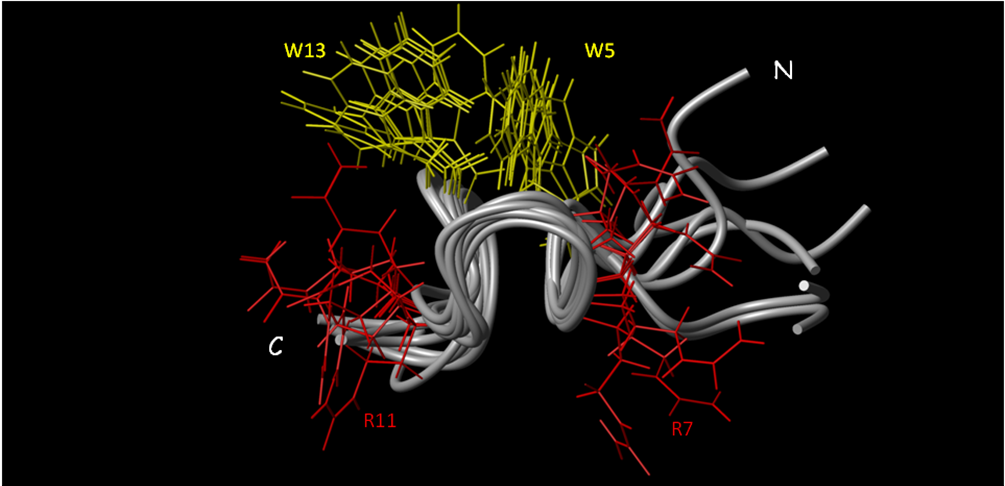


**Figure S10**: Superposed backbone traces for the NMR-derived structural ensemble of HPLW at 318 K. The side chain of Trp5, Arg7, Arg11 and Trp13 are represented as neon.

**Table S1**: ^1^H chemical shift assignment of the HPLW peptide at 318 K.

| **Residue** | **Atom** | **δ(ppm)** |
| --- | --- | --- |
| **K1** | H | 8.32 |
| **K1** | HA | 4.36 |
| **K1** | HZ1 | 7.43 |
| **K1** | HZ2 | 6.78 |
| **Q2** | H | 8.75 |
| **Q2** | HA | 4.36 |
| **Q2** | QB | 1.89 |
| **Q2** | QG | 2.24 |
| **L3** | H | 8.50 |
| **L3** | HA | 4.40 |
| **L3** | QB | 1.51 |
| **L3** | HG | 1.32 |
| **L3** | QD2 | 0.75 |
| **L4** | H | 8.63 |
| **L4** | HA | 4.42 |
| **L4** | QB | 1.42 |
| **L4** | HG | 1.29 |
| **L4** | QQD | 0.69 |
| **W5** | H | 8.50 |
| **W5** | HA | 4.77 |
| **W5** | HB2 | 3.08 |
| **W5** | HB3 | 2.98 |
| **W5** | HD1 | 7.09 |
| **W5** | HE3 | 6.47 |
| **W5** | HE1 | 10.27 |
| **W5** | HZ3 | 6.35 |
| **W5** | HZ2 | 7.37 |
| **W5** | HH2 | 7.03 |
| **I6** | H | 8.29 |
| **I6** | HA | 4.26 |
| **I6** | HB | 1.56 |
| **I6** | HG13 | 0.93 |
| **I6** | HG12 | 1.20 |
| **I6** | QD1 | 0.71 |
| **R7** | H | 8.12 |
| **R7** | HA | 4.28 |
| **R7** | HB2 | 1.58 |
| **R7** | HB3 | 1.48 |
| **R7** | QG | 1.39 |
| **R7** | QD | 2.85 |
| **R7** | HH21 | 6.95 |
| **R7** | HH22 | 6.68 |
| **S8** | H | 8.63 |
| **S8** | HA | 4.45 |
| **S8** | QB | 3.81 |
| **G9** | H | 8.85 |
| **G9** | QA | 3.91 |
| **D10** | H | 8.18 |
| **D10** | HA | 4.56 |
| **D10** | QB | 2.68 |
| **R11** | H | 8.25 |
| **R11** | HA | 4.43 |
| **R11** | QB | 1.48 |
| **R11** | QD | 2.89 |
| **R11** | HH21 | 7.16 |
| **R11** | HH22 | 6.62 |
| **P12** | HA | 4.54 |
| **P12** | QB | 1.45 |
| **P12** | QG | 1.790 |
| **P12** | QD | 3.33 |
| **W13** | H | 8.33 |
| **W13** | HA | 4.71 |
| **W13** | HB2 | 3.13 |
| **W13** | HB3 | 2.98 |
| **W13** | HD1 | 7.16 |
| **W13** | HE3 | 7.45 |
| **W13** | HE1 | 10.42 |
| **W13** | HZ3 | 7.05 |
| **W13** | HZ2 | 7.35 |
| **W13** | HH2 | 7.20 |
| **Y14** | H | 7.56 |
| **Y14** | HA | 4.68 |
| **Y14** | HB2 | 2.56 |
| **Y14** | HB3 | 2.31 |
| **Y14** | QD | 7.81 |
| **Y14** | QE | 6.67 |
| **Y15** | H | 7.61 |
| **Y15** | HA | 4.47 |
| **Y15** | HB2 | 2.78 |
| **Y15** | HB3 | 2.69 |
| **Y15** | QD | 6.95 |
| **Y15** | QE | 6.73 |
| **T16** | H | 8.46 |
| **T16** | HA | 4.37 |
| **T16** | HB | 4.16 |
| **T16** | QG2 | 1.02 |
| **S17** | H | 8.29 |
| **S17** | HA | 4.42 |
| **S17** | QB | 3.81 |

**Table S2**: Observed and calculated average hydrogen bond lengths for HPLW at 298K

| **Number** | **Residue** | **Atom** | **Number** | **Residue** | **Atom** | **Distance**  **(Å)** |
| --- | --- | --- | --- | --- | --- | --- |
| 5 | TRP | HN | 13 | TRP | O | 1.99 |
| 10 | ASP | HN | 7 | ARG | O | 2.00 |
| 13 | TRP | HN | 5 | TRP | O | 1.95 |
| 15 | TYR | HN | 3 | LEU | O | 2.09 |
| 17 | SER | HN | 15 | TYR | O | 1.95 |

**Table S3**: Observed and calculated average hydrogen bond lengths for HPLW at 318K

| **Number** | **Residue** | **Atom** | **Number** | **Residue** | **Atom** | **Distance**  **(Å)** |
| --- | --- | --- | --- | --- | --- | --- |
| 9 | GLY | HN | 7 | ARG | O | 1.96 |
| 10 | ASP | HN | 7 | ARG | O | 2.60 |
